# Telestroke: Maintaining Quality Acute Stroke Care During the COVID-19 Pandemic

**DOI:** 10.1089/tmj.2021.0149

**Published:** 2022-04-19

**Authors:** Theresa Sevilis, Mark McDonald, Amanda Avila, Gregory Heath, Lan Gao, Gayle O'Brien, Mohammed Zaman, Adam Heller, Muhammad Masud, Nima Mowzoon, Thomas Devlin

**Affiliations:** ^1^ TeleSpecialists, LLC, Fort Myers, Florida, USA.; ^2^ Public Health Department, University of Tennessee-Chattanooga, Chattanooga, Tennessee, USA.; ^3^ Mathematics Department, University of Tennessee-Chattanooga, Chattanooga, Tennessee, USA.; ^4^ Department of Neurology, University of Tennessee Health Science Center, Memphis, Tennessee, USA.

**Keywords:** telestroke, COVID-19, quality, telemedicine, telehealth

## Abstract

**Introduction:**

The coronavirus disease 2019 (COVID-19) pandemic has significantly impacted acute stroke care globally. Decreased stroke presentations and concern for delays in acute stroke care have been identified. This study evaluated the impact of COVID-19 on the timely treatment of patients with thrombolytics at hospitals utilizing telestroke acute stroke services.

**Methods:**

Acute stroke consultations seen in 171 hospitals (19 states) via telestroke from December 1, 2019, to June 27, 2020, were extracted from the TeleCare™ database. The consults were divided into pre-COVID and COVID groups (March 15, 2020, start of COVID group). The consults were reviewed for age, sex, hospital, state, date seen, last known normal, arrival time, consult call time, needle time, thrombolytic candidate, and National Institutes of Health Stroke Scale (NIHSS) score. The total number of consults, median door to needle (DTN) time for emergency department (ED) patients, and call to needle (CTN) time for inpatients were calculated.

**Results:**

Pre-COVID, 15,226 stroke consults were evaluated compared with 11,105 in the COVID group, a 27% decrease. Pre-COVID, 1,071 ED patients (7.9%) received thrombolytics and 66 inpatients (4.0%), while COVID, 813 ED patients (8.2%) and 70 inpatients (5.7%). The median DTN time for ED patients pre-COVID was 42 (32, 55) versus 40 (31, 52) in the COVID group, with no statistically significant difference between groups. CTN time pre-COVID was 53 (35, 67) versus 46 (35, 61) in the COVID group, with no statistically significant difference between groups.

**Conclusions:**

Telestroke assessments allowed for uninterrupted acute stroke care and treatment stability despite nursing and other resource realignments triggered by the COVID-19 pandemic.

## Introduction

The coronavirus disease 2019 (COVID-19) pandemic has globally impacted stroke care with reports of significant decreases in patients who presented with stroke symptoms and received acute treatments. It is suspected that the decline in patient presentations was related to social distancing and fear of seeking treatment, with average delays in presentation reported up to 160 min.^1–3^ The delay in presentation led to reduced treatment given the limited time window for interventions. Other factors contributing to the reported treatment decline were delays related to new safety procedure and staffing shortages/reallocation. Many telestroke systems were already in place to assist with acute stroke care before the COVID-19 pandemic. These programs did not have to face the challenges of implementing telemedicine during a pandemic allowing for additional safety for patients and staff in their current care model.

While the World Health Organization declared COVID-19 to be a pandemic on March 11, 2020, it was not until March 14–16, 2020, that many U.S. states started to issue stay–at-home orders or travel bans. These restrictions led to reduced volumes of patients presenting with stroke symptoms, with reports ranging from a 20% to 50% decrease.^4–6^ The ability to provide fast and efficient acute stroke care to those who were presenting was a major concern as 2–6% of COVID-19 patients requiring hospitalization were reported to have strokes.^7^

While there have been reports of reduced acute stroke treatment, there are limited data available on the impact of the COVID-19 pandemic on door to needle (DTN) times and more specifically the role telestroke plays. One study by Huan et al. described a 50.8% decrease in stroke diagnoses, no significant difference in stroke severity, and a lower rate of treatment in their telestroke network.^6^ There have been reports of some facilities maintaining DTN times without mention of any role of telestroke services.^2,8^

In this study, we evaluated the impact of the COVID-19 pandemic on the ability to provide timely acute stroke care via previously well-established telestroke programs. We hypothesized that protocols in place would rapidly adapt to maintain quality acute stroke care during a time of crisis throughout the health care system.

## Methods

The data that support the findings of this study are available from the corresponding author upon reasonable request. This retrospective study was approved by Western Institutional Review Board, and informed consent was waived by the Institutional Review Board.

Acute stroke consultations seen by Telespecialists, LLC physicians in 171 hospitals located in 19 states via telestroke from December 1, 2019, to June 27, 2020, were extracted from the Telecare™ database. The acute stroke consultations consisted of a hospital activating a stroke alert and calling a telemedicine call center for stroke symptoms within 24 h of last known normal (LKN). Once activated, a neurologist goes direct to videoconferencing without any prescreening process. The consults were divided into two groups of 15 weeks with the pre-COVID group including consults seen from December 1, 2019, to March 14, 2020, and the COVID group including consults seen from March 15, 2020, to June 27, 2020. The consults were reviewed for the following: age, sex, hospital, state, date seen, LKN, arrival time, consult call time, needle time, thrombolytic candidate, and National Institutes of Health Stroke Scale (NIHSS) score.

Each of the 171 hospitals was supported by a quality management system (QMS) that creates tailored program goals incorporating best stroke practices, defined metrics, identification of specific process improvement plans, and follow through with root cause analysis and countermeasures when appropriate. A QMS dyad team consisting of a registered nurse stroke coordinator and a neurologist met bimonthly or monthly with delegates from the hospital (typically the facility's stroke coordinator) to monitor performance, identify opportunities for improvement in the stroke alert protocols, and implement process improvement plans.

The total number of consults seen in the two groups was compared and the percentage change calculated. The percentage of consults assessed in the emergency department (ED) and inpatient who received thrombolytics was calculated for each group. The median DTN time for ED consults and call to needle (CTN) for inpatient consults, defined as the time the stroke alert was called to the telemedicine call center for thrombolytic administration, were calculated. The median NIHSS score within each group was calculated as well as the median NIHSS score for patients receiving thrombolytics within each group. The average LKN to arrival was calculated for ED patients. The mean age of patients in each group was calculated.

Descriptive statistics are presented as counts and percentages for categorical variables. Means and standard deviations, and median and interquartile range are reported for continuous variables. Characteristics of pre-COVID and COVID group consults were compared using the Mantel–Haenszel χ^2^ test and odds ratios with 95% confidence limits for categorical variables, while comparisons for continuous variables were assessed using the independent samples median test.

All tests of statistical significance were conducted using a two-sided type I error of 5%. All analyses were carried out in either SPSS version 26 (IBM Corporation) or in R version 4.0.1 (The R Foundation).

## Results

In the pre-COVID group, 15,226 stroke consults (13,566 ED and 1,660 inpatient) were evaluated compared with 11,105 in the COVID group (9,870 ED and 1,235 inpatient). No consults were excluded from the data sets. There was a 27% decrease in stroke consults. There was no significant difference in age, sex, or median NIHSS score seen between the two groups (*Table 1*).

**Table 1.** Consult Demographic Characteristics

	PRE-COVID	COVID	*p*
Total cases	15,226	11,105	N/A
Age (years)	67.0 ± 15.8	66.7 ± 15.8	0.233
Female sex (%)	8,082 (53.1)	5,802 (52.2)	0.067
Consult in ED	13,566 (89.1)	9,870 (88.9)	0.288
Consult inpatient	1,660 (10.9)	1,235 (11.1)	0.288
Median NIHSS score	2 (0, 5)	2 (0, 5)	0.492

Data presented as *n* (%) except for age, which is mean, and NIHSS, which is median (IQR).

COVID, coronavirus; ED, emergency department; IQR, interquartile range; NIHSS, National Institutes of Health Stroke Scale.

In the pre-COVID group, 1,071 ED consults (7.9%) received thrombolytics and 66 inpatient consults (4.0%) compared with the COVID group with 813 ED consults (8.2%) and 70 inpatient consults (5.7%). There was no significant increase in the ED consults treated with thrombolytics (*p* = 0.443), but there was a significant increase in inpatient treatment with thrombolytics (*p* = 0.033) (*Table 2*).

**Table 2.** Outcomes

	PRE-COVID	COVID	*p*
Received thrombolytics total (%)	1,137 (7.5)	883 (8.0)	0.797
ED thrombolytics (%)	1,071 (7.9)	813(8.2)	0.443
Inpatient thrombolytics (%)	66 (4.0)	70 (5.7)	0.033^a^
ED DTN time	42 (32, 55)	40 (31, 52)	0.154
IP CTN time	53 (35, 67)	46 (35, 61)	0.177
Median NIHSS score	5 (3, 11)	5 (3, 10)	0.201

Data presented as *n* (%) except for NIHSS score, ED DTN time, and IP CTN time, which is median (IQR).

^a^ Statistical significance at *p* < 0.05.

CTN, call to needle; DTN, door to needle; IP, inpatient.

There was no statistically significant difference among the group demographics. Demographic information was not available for all patients. There were 226 consults excluded from age analysis due to unavailable data, and 544 consults were excluded from NIHSS score analysis due to unavailable data.

**Fig. 1.**
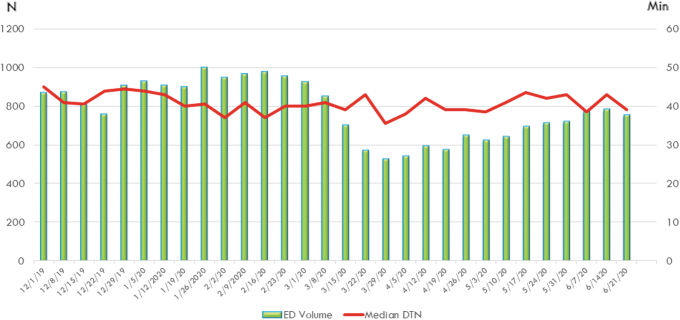
ED volumes per week and median DTN times per week. Weekly volumes of acute stroke consults in the ED in 171 hospitals in 19 states, and the average DTN time in minutes for that week of the consults who received thrombolytics. DTN, door to needle; ED, emergency department.

**Fig. 2.**
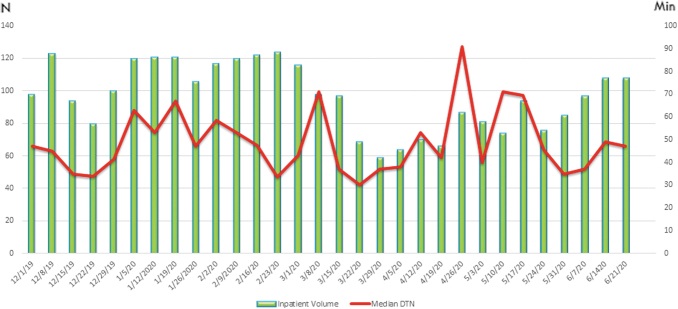
Inpatient acute stroke consult volume per week and median CTN times per week. Weekly volumes of acute stroke consults seen inpatient in 171 hospitals in 19 states, and the average CTN time in minutes for that week of the consults who received thrombolytics. CTN, call to needle.

The median DTN time for ED consults in the pre-COVID group was 42 mins (32, 55) versus 40 (31, 52) in the COVID group, with no statistically significant difference between groups, *p* = 0.15 (*Table 2* and *Fig. 1*). The CTN in the pre-COVID group was 53 (35, 67) versus 46 (35, 61) in the COVID group, with no statistically significant difference between groups, *p* = 0.18 (*Table 2* and *Fig. 2*). No consults were excluded from the DTN or CTN analysis.

The median LKN to arrival time for all consults with available data in the pre-COVID group (LKN was not available or unknown for 2,119 consults) was 157.7 min, and in the COVID group (LKN was not available or unknown for 1,952 consults) was 154.0 min, with no statistically significant difference, *p* = 0.478. The median LKN to arrival time in the Pre-COVID group (15 patients who were excluded due to not clearly documented LKN) for consults who received thrombolytics was 61.0 min, and in the COVID group (six patients who were excluded due to unclear documentation of LKN) was 69.0 min, which was not a statistically significant difference, *p* = 0.091. The median LKN to arrival time in the pre-COVID group (2,104 consults with unknown LKN or unavailable data were excluded) for consults who did not receive thrombolytics was 184.0 min, and in the COVID group (1,946 consults with unknown LKN or unavailable data were excluded) was 185.0 min, which was not statistically significant, *p* = 0.101.

## Discussion

The clinically significant finding of this study was that utilizing an established, quality-focused telestroke service allowed for maintained acute stroke treatment with thrombolytics during the COVID-19 pandemic. While the COVID-19 pandemic led to a significant decline in stroke presentations globally, there was no significant difference in the percentage of consults who received thrombolytics in our cohort. The decline in consult volume of 27% was consistent with reports from other publications.^4–6^ With no change in treatment percentage and presenting NIHSS scores, there is no indication that patients with more mild symptoms were staying home, while those with more severe symptoms presenting as has been speculated.

The ability to maintain a consistent DTN time in the setting of additional safety screenings, staffing shortages, and reallocation was likely multifactorial. With an established telestroke service line, a QMS in place, and dedicated staff committed to maintaining quality of care, there were ample resources to help assist hospitals with the workflow and protocol changes necessary to maintain or continue to improve a stroke program. A neurologist evaluation via telemedicine also allowed for potential decrease in time to bedside, given that no personal protective equipment (PPE) or travel throughout the hospital was required. This decrease in bedtime response is also consistent overnight when a neurologist might otherwise not be in-house.

The increase in percentage of inpatient stroke consults who received thrombolytics was potentially a result of a higher acuity level of hospitalized patients with increased risk factors for stroke. It was unknown in this study if the patients had COVID-19, and therefore, a direct relationship to the disease cannot be made.

This study highlights the utility of telestroke services in maintaining timely, high-quality acute stroke care even when the health care system is under the significant stress of a global public health crisis. Further studies of the impact of telestroke on timely treatment with neurointervention procedures would be of interest to evaluate the role of telestroke in maintaining quality throughout the spectrum of acute stroke care. Further research on the outcomes of telestroke consults versus in-person consults would also be of interest.

There were a few limitations to this study. The data were collected from only initial stroke consults and the final diagnosis from the admission was unknown. Another limitation was that the data were entered into the database by the neurologists, but this was mitigated as all DTN and CTN times verified with facilities. The COVID-19 status of the consults was not known, and therefore, no direct effect of the disease on stroke could be evaluated.

## Conclusions

Utilization of a telestroke program in close collaboration with an established QMS was associated with preserved DTN times and allowed for uninterrupted acute stroke care despite nursing and other local resource realignments triggered by the COVID-19 pandemic.
